# Epithelial–myoepithelial carcinoma of the nasopharynx: A case report and review of the literature

**DOI:** 10.3389/fonc.2022.923579

**Published:** 2022-08-05

**Authors:** Wei Zhang, Xiao-xiao Wang, Xiao-li Wang, Yan Zhang, Xiu-feng Li, Yang Li, Yuan-yuan Cai, Hui-qi Ren, Yun-xiang Zhang, Fu-rong Hao

**Affiliations:** ^1^ Clinical School, Weifang Medical University, Weifang, China; ^2^ Department of Radiation Oncology, Weifang People’s Hospital, Weifang, China; ^3^ Department of Pathology, Weifang People’s Hospital, Weifang, China; ^4^ Weifang Key Laboratory of Radiophysics and Oncological Radiobiology, Weifang, China

**Keywords:** epithelial–myoepithelial carcinoma, nasopharynx, immunohistochemistry, concurrent chemoradiotherapy, case report

## Abstract

**Background:**

Epithelial–myoepithelial carcinoma (EMCa) is a rare low-grade malignant tumor that most commonly occurs in the salivary glands, with approximately 320 cases having been reported worldwide. Here, we report the third case of EMCa occurring in the nasopharynx. Rare cases in the breast, pituitary gland, lacrimal gland, nose, paranasal sinus, nasal cavity, trachea and bronchus, lung, and even the pleura mediastinalis have also been reported. Histopathology and immunohistochemistry are useful for confirming the diagnosis of EMCa, which is characterized by biphasic tubular structures composed of inner ductal and outer clear myoepithelial cells and stains for different markers in each layer. However, because of the rarity of EMCa, the clinicopathological characteristics and treatment of these patients remain unclear.

**Case presentation:**

We report a rare case of EMCa of the nasopharynx. A 51-year-old man presented with a 5-month history of pain while swallowing and aggravation accompanied by right ear tinnitus lasting for 1 month. Nasopharyngoscopy and magnetic resonance imaging (MRI) of the nasopharynx and neck revealed a 5.6 cm × 3.4 cm × 3.1 cm mass in the nasopharyngeal space, invasion of the right cavernous sinus, and lymph node enlargement in the right retropharyngeal space. On 17 April 2019, based on the histopathological and immunohistochemical features, a final diagnosis of EMCa of the right nasopharynx was made. The patient underwent concurrent chemoradiotherapy (CCRT), and his symptoms were relieved after treatment. On 10 January 2022, nasopharynx MRI and biopsy revealed local recurrence, but chest and abdominal computed tomography (CT) showed no obvious signs of metastasis. The local recurrence-free survival (LRFS) period was 33 months.

**Conclusion:**

To the best of our knowledge, this is the third reported case of EMCa in the nasopharynx and the only case of EMCa in the nasopharynx treated with CCRT, and a partial response was achieved. Therefore, to improve the quality of life and prognosis of patients with unresectable tumors, we believe that CCRT is a suitable option. Further clinical observations are required to elucidate the pathophysiology and prognosis of EMCa.

## Background

Epithelial–myoepithelial carcinoma (EMCa) is a rare low-grade malignant epithelial neoplasm composed of variable proportions of ductular cells with large, clear cytoplasmic myoepithelial cells arranged around the periphery of the ducts (1–4). EMCa predominantly arises from the parotid gland, accounting for less than 1% of all salivary gland tumors and approximately 2% of malignant salivary gland neoplasms (3–12). Of all the types of nasopharyngeal malignancies treated at our center, the incidence of EMCa is 1/315 (0.3%) as of 2021. This tumor can occur in unusual sites, such as the breast (13–15), pituitary gland (16), paranasal sinus (9, 17, 18), lacrimal gland (19–22), nasal cavity (1, 3, 6, 23–25), trachea and bronchus (26, 27), lung (28–34), nasopharynx (4, 5), and even the pleura mediastinalis (35). EMCa was first reported by Donath in 1972, and 8 patients have been reported with a salivary gland tumor that was termed EMCa (36). However, it was described in the literature as early as 1956 (2, 7, 37). Only approximately 320 cases have been reported thus far (2, 11, 38). The domestic and foreign literature mostly consist of case reports, with two cases of nasopharyngeal EMCa being reported by Imate et al. in 2000 (5) and Kim et al. in 2015 (4). Here, we report the third case of EMCa of the nasopharynx in a 51-year-old man who was treated with concurrent chemoradiotherapy (CCRT).

## Case presentation

**Figure 1:**
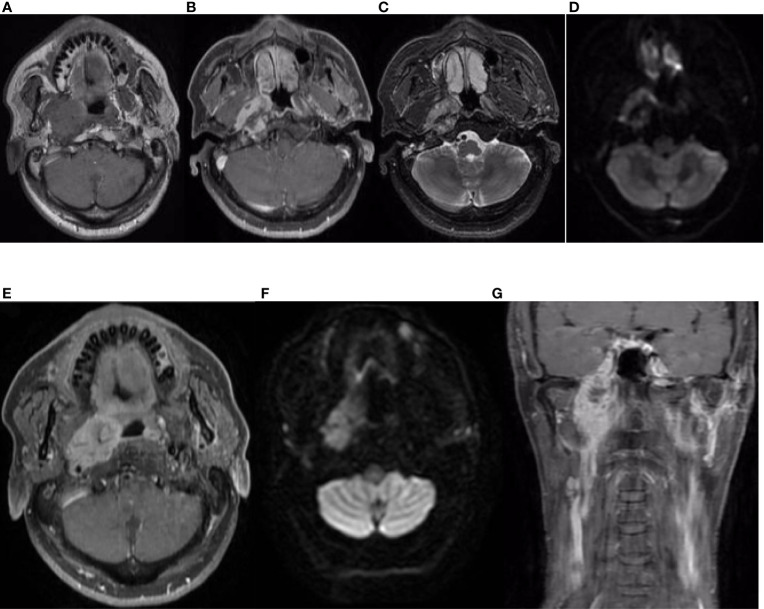
Nasopharynx and neck MRI findings from 15 April 2019. **(A–D)** The axial planes of T1-weighted, enhanced T1-weighted, T2-weighted, and diffusion-weighted imaging (DWI) MRI of the nasopharynx, showing a 5.6 cm × 3.4 cm × 3.1 cm mass within the right parietal and lateral walls of the nasopharynx, accompanied by skull base bone invasion, invasion of the oropharyngeal lateral wall and right parapharyngeal space, the right medial pterygoid muscle, and the musculus longus capitis. **(E, F)** The axial planes of enhanced T1-weighted DWI MRI revealed enlargement of the right retropharyngeal lymph node. **(G)** The coronal plane of enhanced T1-weighted imaging showed tumor involvement with the cavernous sinus.

**Figure 2:**
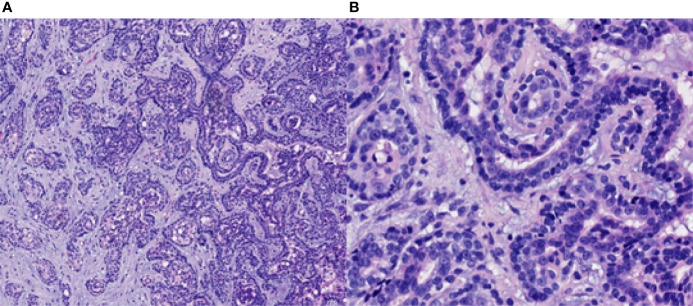
Pathological findings of the nasopharyngeal biopsy obtained on 17 April 2019. The EMCa of the nasopharynx was mainly composed of an inner layer of epithelial cells and an outer layer of clear cytoplasmic myoepithelial cells. H&E staining; magnification, ×10 **(A)** and ×40 **(B)**. EMCa, epithelial–myoepithelial carcinoma; H&E, hematoxylin and eosin.

On 14 April 2019, a 51-year-old man presented with a 5-month history of pain while swallowing and aggravation accompanied by right ear tinnitus lasting for 1 month. He was admitted to the otolaryngology department of our hospital. Nasopharyngoscopy revealed a mass on the right nasopharyngeal wall, and a partial tissue sample was obtained *via* biopsy. Magnetic resonance imaging (MRI) of the nasopharynx and neck on 15 April 2019 revealed a 5.6 cm × 3.4 cm × 3.1 cm mass within the right parietal and lateral walls of the nasopharynx, accompanied by skull base bone erosion, and invasion of the oropharyngeal lateral wall and right parapharyngeal space, the right medial pterygoid muscle and musculus longus capitis, and the cavernous sinus and the posterior nostril. Simultaneously, lymph node enlargement was found in the right retropharyngeal space (**Figure 1**). A final diagnosis of EMCa was made based on the histological and immunohistochemical (IHC) features of nasopharyngeal biopsy on 17 April 2019. Hematoxylin and eosin staining showed a classical biphasic pattern with typical epithelial cells and myoepithelial cells (**Figure 2**) without nerve invasion or vascular cancer embolus. The patient was transferred to our department for further treatment. The tumor was staged as IVa (cT4N1M0) according to the 8th edition of the AJCC staging system (39).

## Treatment and outcomes

**Figure 3:**
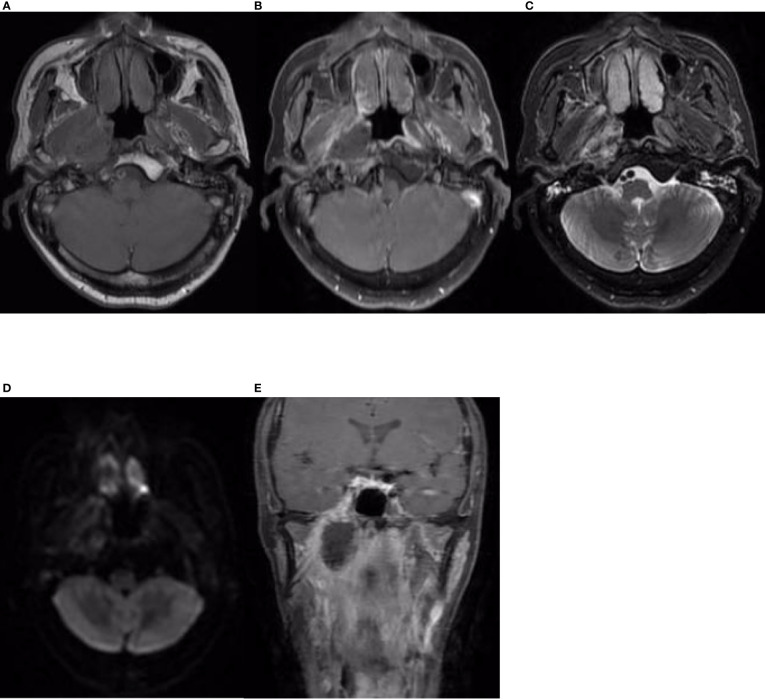
Nasopharynx and neck MRI findings from 29 July 2019. **(A–D)** The axial planes of T1-weighted, enhanced T1-weighted, T2-weighted, and DWI and **(E)** the coronal plane of enhanced T1-weighted MRI, showing that the nasopharyngeal lesions on the right side subsided significantly, but there was a 4.8 cm × 3.0 cm × 2.7 cm residual area of low signal and no enhancement, which suggested necrosis.

**Figure 4:**
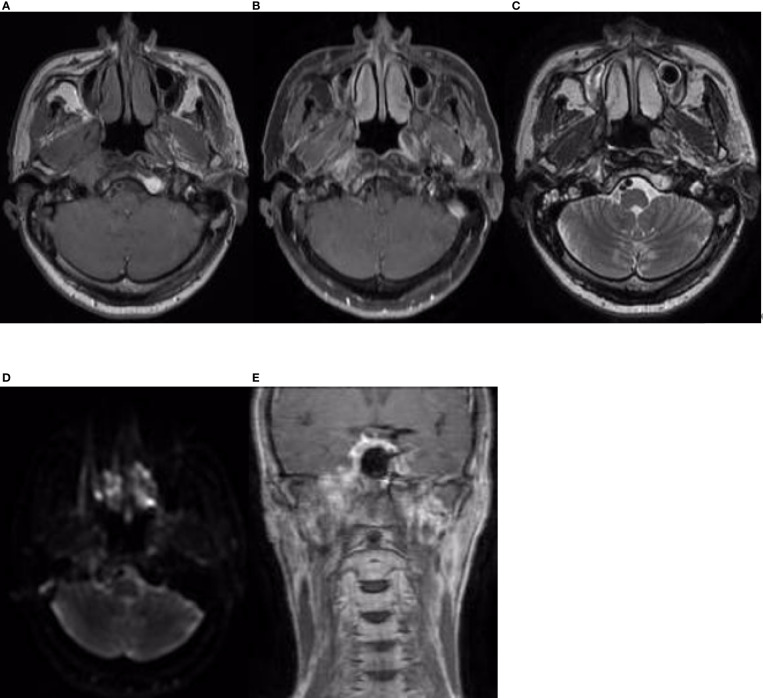
Nasopharynx and neck MRI findings from 7 July 2020. **(A–D)** The axial planes of T1-weighted, enhanced T1-weighted, T2-weighted, and DWI and **(E)** the coronal plane of enhanced T1-weighted MRI, showing that the nasopharyngeal lesions and the necrotic area had subsided significantly. However, there was still a residual area of inhomogeneous enhancement in the right nasopharynx and parapharyngeal space, demonstrating restricted diffusion on DWI.

**Figure 5:**
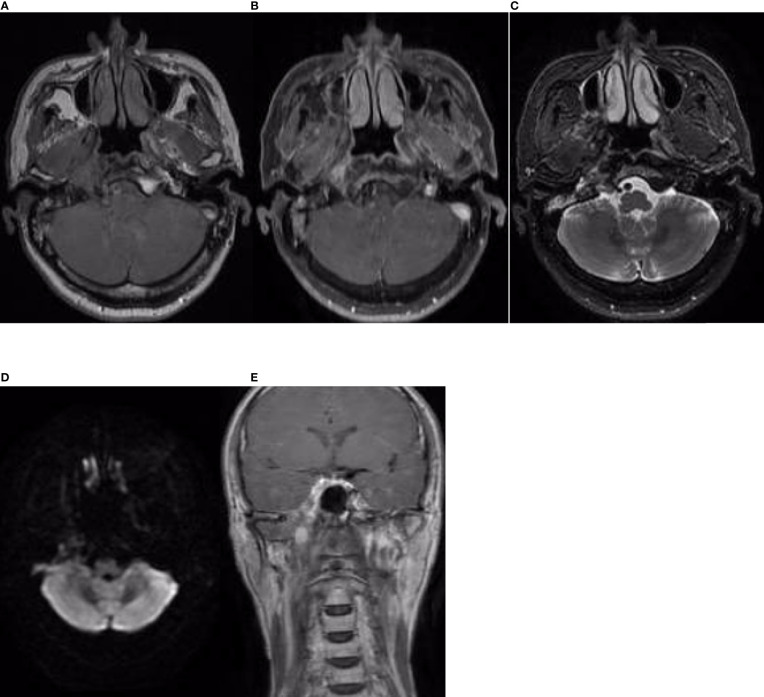
Nasopharynx and neck MRI findings from 27 April 2021. **(A–D)** The axial planes of T1-weighted, enhanced T1-weighted, T2-weighted, and DWI and **(E)** the coronal plane of enhanced T1-weighted MRI, showing that the residual area of inhomogeneous enhancement had shrunk.

**Figure 6:**
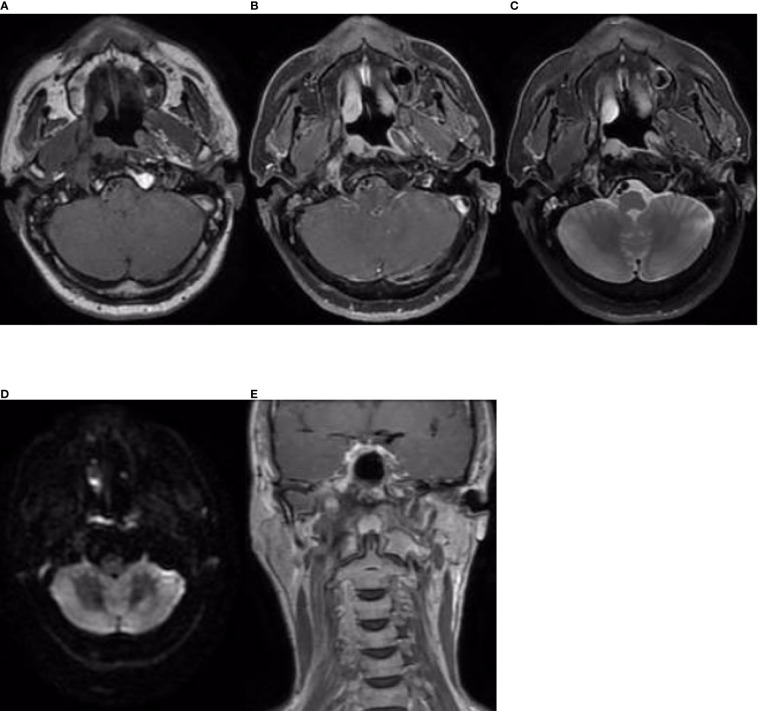
Nasopharynx and neck MRI findings from 10 January 2022. **(A–D)** The axial planes of T1-weighted, enhanced T1-weighted, T2-weighted, and DWI and **(E)** the coronal plane of enhanced T1-weighted MRI, showing an area of enhancement in the right nasopharyngeal and parapharyngeal space. DWI demonstrated slightly restricted diffusion, which was considered indicative of recurrence.

**Figure 7:**
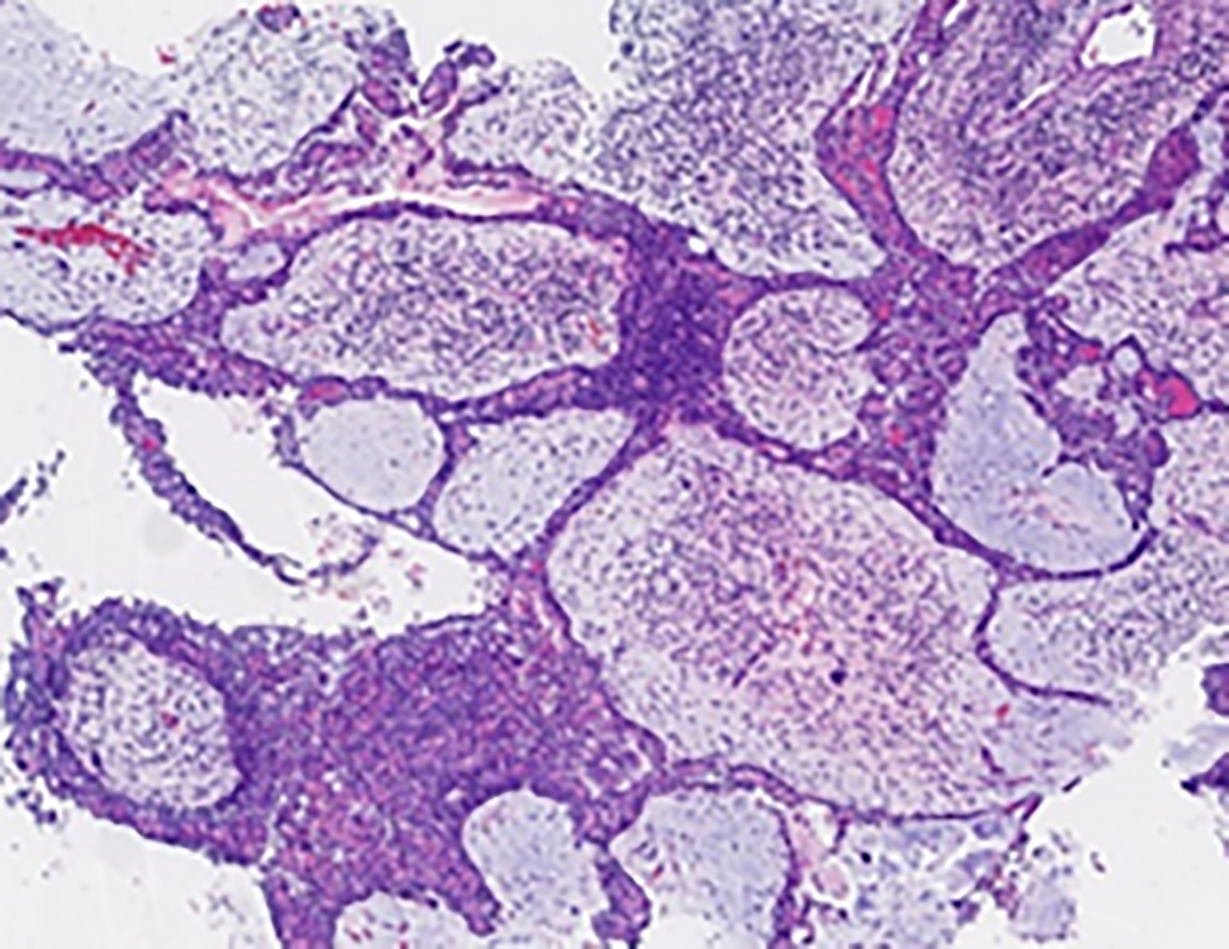
Pathological findings of the nasopharyngeal biopsy obtained on 12 January 2022. Nasopharyngeal EMCa recurrence was considered. H&E staining; magnification, ×4. EMCa, epithelial–myoepithelial carcinoma; H&E, hematoxylin and eosin.

The patient received CCRT in our department. Target volumes were delineated according to the consensus (40), and intensity-modulated radiation therapy (IMRT) was performed from 24 April 2019 to 10 June 2019. Over a total of 33 fractions, a dose of 7,000 cGy was delivered to the tumor, and a dose of 6,800 cGy was delivered to the metastatic lymph node in the right retropharyngeal space; the dose delivered to high-risk regions was 6,006 cGy, referred to as clinical target volume 1 (CTV1), and the dose delivered to low-risk regions was 5,775 cGy, referred to as CTV2. During radiation therapy, 80 mg/m^2^ cisplatin was administered every 3 weeks for up to 3 cycles. On 29 July 2019, 1.5 months after the end of treatment, nasopharynx and neck MRI revealed that the nasopharyngeal lesions on the right side had shrunk significantly, but there was a 4.8 cm × 3.0 cm × 2.7 cm residual area of low signal and no enhancement, which suggested necrosis (**Figure 3**). In September 2019, the patient suffered from massive nasopharyngeal hemorrhage at home, which improved after receiving symptomatic treatment such as hemostasis in a local hospital. Then, follow-up of the patient became irregular. On 7 July 2020, 1 year after the end of treatment, nasopharynx and neck MRI revealed that the nasopharyngeal lesions had subsided significantly, but there was still an area of inhomogeneous residual enhancement of the right nasopharynx and parapharyngeal space (**Figure 4**). The residual area of inhomogeneous enhancement had shrunk to a smaller size, and there were no obvious signs of recurrence or metastasis on reexamination on 27 April 2021 (**Figure 5**). The patient’s pain while swallowing was completely relieved, and his symptoms of right tinnitus were partially relieved. On 10 January 2022, the patient was examined at our department, and nasopharynx and neck MRI revealed that the nasopharyngeal enhancement area was more obvious than before, which was considered recurrence (**Figure 6**). Chest and abdominal computed tomography (CT) showed no obvious signs of metastasis. Nasopharyngeal biopsy was performed that same day, and the pathological (**Figure 7**) and IHC findings on 18 January 2022 suggested EMCa recurrence of the nasopharynx. In summary, the follow-up at 33 months revealed recurrence but no distant metastasis.

## IHC findings

**Figure 8:**
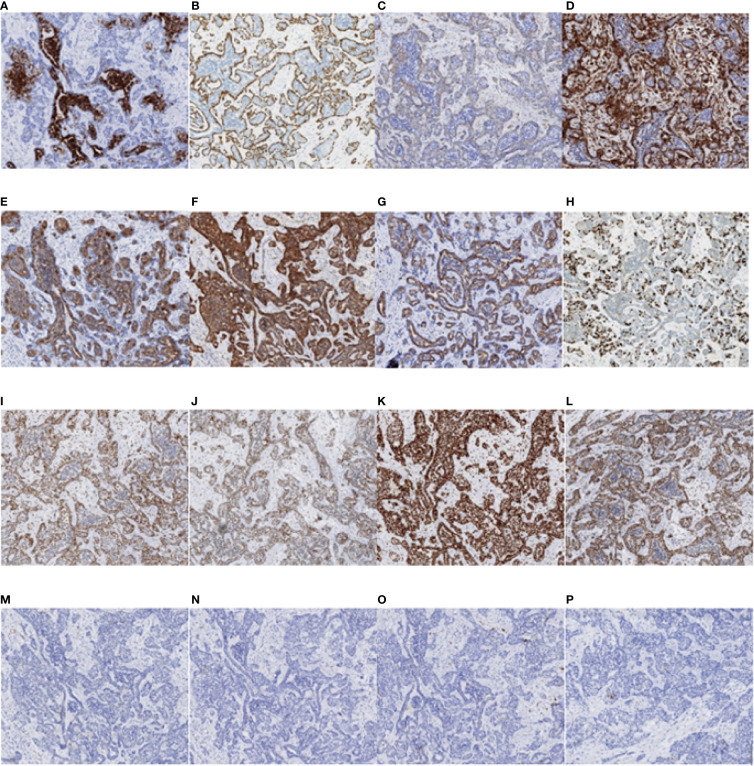
Immunohistochemistry test results of the nasopharyngeal biopsy obtained on 17 April 2019. **(A)** CK7 positivity was observed in epithelial cells. **(B–D)** P63, SMA, and VIM positivity was observed in myoepithelial cells. **(E–G)** CK, EGFR, and CD117 positivity was observed in tumor cells. **(H)** Ki-67 positivity was observed in 35% of the tumor cells. **(I–L)** MLH1, MSH2, MSH6, and PMS2 positivity was observed in tumor cells. **(M–O)** Actin, GFAP, and S-100 negativity was observed in tumor cells. **(P)** PD-L1 positivity was observed in less than 1% of tumor cells and in 10% of stromal cells (magnification, ×10). CK, cytokeratin; SMA, smooth muscle actin; VIM, vimentin; EGFR, epidermal growth factor receptor; GFAP, glial fibrillary acidic protein; PD-L1, programmed death ligand-1.

On 17 April 2019, at first admission, IHC staining revealed that cytokeratin (CK) was widely positive, and the inner epithelial cells were positive for CK7, an epithelial cell marker. The outer myoepithelial cells were positive for P63, smooth muscle actin (SMA) and vimentin (VIM), consistent with a myoepithelial phenotype, confirming the diagnosis of EMCa. Epidermal growth factor receptor (EGFR) and CD117 staining was also positive, while S-100, actin, and glial fibrillary acidic protein (GFAP) staining was negative. The expression of programmed death ligand-1 (PD-L1) was less than 1% in tumor cells and 10% in stromal cells. The expression of MLH1, MSH2, MSH6, and PMS2 and all four mismatch repair (MMR) proteins was positive, which was interpreted as proficient mismatch repair (pMMR). Ki-67 was positive in 35% of the neoplastic cells (**Figure 8**).

**Figure 9:**
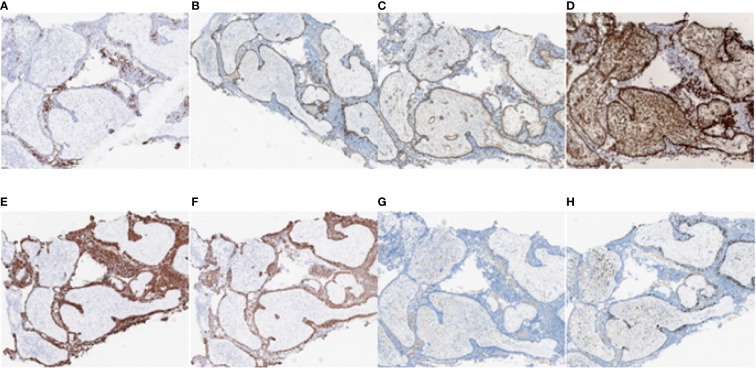
Immunohistochemistry test results of the nasopharyngeal biopsy obtained on 18 January 2022. **(A)** CK7 positivity was observed in epithelial cells. **(B–D)** P63, SMA, and VIM positivity was observed in myoepithelial cells. **(E–G)** CK, CK5/6, and S-100 positivity was observed in tumor cells. **(H)** Ki-67 positivity was observed in 15% of tumor cells (magnification, ×4). CK, cytokeratin; SMA, smooth muscle actin; VIM, vimentin.

On 18 January 2022, at the second admission, IHC staining revealed that CK was wildly positive, and the inner epithelial cells were positive for CK7. The outer myoepithelial cells were positive for P63, SMA, and VIM. S-100 was positive in some cells, and CK5/6 was positive in most cells. Ki-67 was positive in 15% of the neoplastic cells (**Figure 9**). The IHC results of the inner epithelial cells and the outer myoepithelial cells are summarized in **Table 1**.

**Table 1:** Immunohistochemistry findings from 17 April 2019 and 18 January 2022.

Antibody	17 April 2019		18 January 2022		
	Inner cells	Outer cells	Inner cells	Outer cells	
**CK7** **P63**	Positive Negative	Negative Positive	Positive Negative	Negative Positive	
**SMA** **VIM**	Negative Negative	Positive Positive	Negative Negative	Positive Positive	
**CK** **EGFR** **CD117**	Positive Positive Positive	Positive Positive Positive	Positive - -	Positive - -	
**MLH1** **MSH2** **MSH6** **PMS2** **Actin** **GFAP** **S-100** **CK5/6** **KI-67**	Positive Positive Positive Positive Negative Negative Negative - Positive	Positive Positive Positive Positive Negative Negative Negative - Positive	- - - - - - Positive Positive Positive	- - - - - - Positive Positive Positive	

CK, cytokeratin; SMA, smooth muscle actin; VIM, vimentin; EGFR, epidermal growth factor receptor; GFAP, glial fibrillary acidic protein; “-”, this test was not performed.

## Discussion

EMCa is a rare and unique tumor that most commonly occurs in the salivary glands (7, 11, 12). The average age at diagnosis is approximately 60 years old, and the incidence is higher in women than in men, with a ratio of 1.34-2:1 (2, 7). These tumors have a propensity for infiltration but low malignancy (4). Thus, they invade nerves, blood vessels, and bone, and local recurrence is often observed (0–56%) (5), but the mortality rate is low (11). The mean times to recurrence and metastases are 5 years (41) and 15 years (42), respectively. The median disease-free survival (DFS) time is 11.34 years, and the 5- and 10-year disease-specific survival (DSS) rates are 93.5% and 81.8%, respectively (2). Factors associated with overall survival include the following: tumor size < 4 cm, absence of regional nodal or distant metastases, patient age < 80 years at diagnosis, surgical treatment, and Ki-67 index less than 3.5% (7, 10, 43, 44). Age younger than 80 years was the only factor associated with a good prognosis in the case reported herein. Clinical manifestations of EMCa are usually nonspecific and can vary depending on the site of origin and extent of the tumor (10). Tumors involving the nasopharynx may cause symptoms such as nose blockage, epistaxis, tinnitus, hearing loss and pain in the ear (4, 5). In our case, the patient presented with pain while swallowing and tinnitus, for which the main cause was the lesion occupying the surrounding tissues.

The confirmative diagnosis of EMCa depends on the histopathological and IHC results (11). EMCa exhibits a biphasic histological morphology, with the inner layer being a single layer of cubic sacral epithelial cells and the peripheral layer being composed of clear cytoplasmic myoepithelial cells, with a duct-like structure and infiltrating margins (7, 11). Compared with the low specificity of radiologic imaging, immunohistochemistry is useful for distinguishing EMCa, as it can depict the characteristic biphasic epithelial–myoepithelial phenotype and the differential staining of markers in each layer; thus, it plays a major role in the final diagnosis (2, 10). IHC staining of glandular epithelial markers such as CK, epithelial membrane antigen (EMA), and CD117 (11) and myoepithelial markers such as SMA, S-100 protein, Actin, VIM, CK14, GFAP and Calponin, and P63 is observed (45–47). Kawahara et al. suggested that P63 is an especially useful marker of myoepithelial cells with naked nuclei in EMCa (48). Nevertheless, studies have shown that VIM and calponin, especially the latter, are sensitive markers for salivary myoepithelial tumor cells. SMA and P63 are relatively less sensitive than calponin (11, 49, 50). In our case, we observed positive CK7 staining in the inner epithelial cells along with P63, SMA, and VIM in the outer myoepithelial cells.

There is currently no consensus regarding the optimal treatment of this disease, largely due to its rarity (24). For other malignant tumors of the head and neck (51), surgery is the preferred method (11). Studies have shown that the true survival benefit of radiotherapy is unclear (7), but others have argued that it may be effective at preventing local recurrence, particularly for neoplasms with a diameter larger than 4 cm (6, 24, 52). A recent study suggested that the complete remission (CR) rate could be improved by consecutive radiotherapy, and if tumors are deemed primarily unresectable, definitive radiotherapy may be used with or without chemotherapy (44). To the best of our knowledge, two cases of EMCa in the nasopharynx reported in the literature were treated by surgical resection (5), and another was treated with CCRT followed by systemic chemotherapy (4), which resulted in a partial response. In our case, due to the anatomical location and the clinical stage of the patient being locally advanced, the tumor was unresectable; thus, CCRT was suggested.

## Conclusion

Although it is a low-grade malignancy, EMCa should be treated aggressively, as it has a tendency for both local recurrence and distant metastasis. Although the optimal treatment strategy for EMCa remains poorly defined due to its rarity, the present study reports the only case of EMCa in the nasopharynx treated with CCRT, and a partial response was achieved. Therefore, to improve the quality of life and prognosis of patients with unresectable tumors, we believe that CCRT is a suitable option. Further accumulation of cases and long-term follow-up data are needed to elucidate the pathophysiology and prognosis of epithelial–myoepithelial carcinoma.

## Data availability statement

The original contributions presented in the study are included in the article/supplementary material. Further inquiries can be directed to the corresponding authors.

## Ethics statement

The studies involving human participants were reviewed and approved by the Ethics Committee of Weifang People’s Hospital. Written informed consent was obtained from the individual(s) for the publication of any potentially identifiable images or data included in this article.

## Author contributions

WZ, X-XW, X-LW, and YZ drafted the manuscript and performed the literature review. X-FL and Y-XZ retrieved and analyzed pathological and immunohistochemistry information. Y-YC and F-RH performed the chemoradiotherapy in this case. YL and H-QR retrieved the imaging data, and WZ and F-RH performed patient follow-up. F-RH and Y-XZ conceived, designed, and supervised the study. This paper properly credits the meaningful contributions of all coauthors and coresearchers. All authors have been personally and actively involved in substantial work leading to the publication of this paper and take public responsibility for its content.

## Funding

This work was supported by Weifang Science and Technology Development Project (Grant NO. 2019YX003).

## Conflict of interest

The authors declare that the research was conducted in the absence of any commercial or financial relationships that could be construed as a potential conflict of interest.

## Publisher’s note

All claims expressed in this article are solely those of the authors and do not necessarily represent those of their affiliated organizations, or those of the publisher, the editors and the reviewers. Any product that may be evaluated in this article, or claim that may be made by its manufacturer, is not guaranteed or endorsed by the publisher.
